# Substitution of citrate with tissue plasminogen activator (rt-PA) for catheter lock does not improve patency of tunnelled haemodialysis catheters in a randomised trial

**DOI:** 10.1186/s12882-021-02243-y

**Published:** 2021-01-28

**Authors:** Pavlina Richtrova, Jan Mares, Lukas Kielberger, Jan Klaboch, Jaromir Eiselt, Tomas Reischig

**Affiliations:** 1 Department of Internal Medicine I, Faculty of Medicine in Pilsen, Charles University, and Teaching Hospital in Pilsen, Alej Svobody 80, 304 60 Plzen, Czech Republic; 2 Biomedical Centre, Faculty of Medicine in Pilsen, Charles University, Pilsen, Czech Republic

## Abstract

**Backround:**

The study aim was to establish if substitution of citrate with rt-PA for catheter lock once weekly can reduce the incidence of catheter-related blood stream infections (CR-BSI) or improve patency of tunneled haemodialysis catheters.

**Methods:**

All incident patients undergoing insertion of a tunneled haemodialysis catheter were screened and included except those suffering infection or using oral anticoagulation. Study participants were randomized into two arms according to the solution applied as catheter lock: receiving either trisodium citrate (Citra-LockTM 4%) only or rt-PA (Actilyse® 1 mg/ml) on the middle session each week with citrate used on the first and third sessions. The incidence of CR-BSI (confirmed by positive blood culture), catheter non-function (complete obstruction), and malfunction (blood flow < 250 ml/min) was recorded. Statistical significance was tested with ANOVA, post hoc analysis was performed by means of multiple linear regression.

**Results:**

Totally, 18 patients were included and followed during 655 haemodialysis sessions. No episode of CR-BSI was detected while 6 catheter non-functions (0.9% sessions) and 101 malfunctions (15.4% sessions) were recorded. The incidence of both events was equal between the study arms: 4 non-functions and 55 malfunctions in the rt-PA arm and 2 non-functions and 46 malfunctions in the citrate arm (*p* = 0.47 and *p* = 0.24, respectively). Additionally, the mean blood flow achieved did not differ significantly between the arms: 326 ± 1,8 and 326 ± 1,9 ml/min (*p* = 0.95) in rt-PA and citrate arms, respectively. Post hoc analysis identified time elapsed since previous session (β = 0.12, *p* = 0.005) and malfunction on previous session (β = 0.25, *p* < 0.001) as significant factors affecting the occurrence of malfunction. By contrast, the study arm, rt-PA application on previous session, and catheter vintage did not enter the model.

**Conclusion:**

Substitution of citrate with rt-PA for catheter lock does not reduce the incidence of catheter malfunction neither does it affect the blood flow achieved during haemodialysis. Catheter patency is related rather to the time interval between sessions and to previous malfunction (thus probably reflecting undefined individual factors). The incidence of CR-BSI within pre-selected haemodialysis population is sporadic (less than 1 per 4.3 patient years in our sample).

**Trial registration:**

Australian New Zealand Clinical Trials Registry, ACTRN12612000152820. Retrospectively registered 03/02/2012.

## Background

A number of guidelines and initiatives strongly recommend native arteriovenous fistulas (AVF) and discourage the use of catheters for chronic dialysis treatment [1–3]. This fistula first policy is based on evidence from large observational studies showing that the use of AVF is associated with the lowest (and catheters with the highest) risk of death from infection and cardiovascular disease [4, 5]. However, in contrast to this recommendation, the proportion of haemodialysis (HD) patients treated with central venous catheters (CVC) is increasing [6, 7]. It is related to the ageing of the HD population and its polymorbidity. The quality of the vascular system is poor, the creation of AVF is difficult, and the maturation is not successful enough. Up to 60% of AVFs fail immediately after surgery or fail to mature [8, 9]. Noordzij et al. found that female patients and those > 80 years were least likely to start HD with an AVF, according to European renal registries between 2005 and 2009 [6].

An alternative to AVF for chronic HD treatment is tunnelled CVC (tCVC). It can serve as temporary access until the maturation of AVF, or it is definitive option for patients without the possibility for native access. Care for patients with tCVC means, in particular, prevention of exit site infection and catheter-related blood stream infection (CRBSI), and care for catheter patency. In addition to common rules of asepsis, catheter lock solutions are used. These solutions consist of various chemicals and are installed after every HD procedure to tCVC at a volume precisely declared on the catheter. Evidence to guide catheter lock solutions is limited. In recent years, there has been a shift from the use of heparin to citrate solutions [10].

In 2011, Hemmelgarn and colleagues demonstrated a beneficial effect of once-weekly recombinant tissue plasminogen activator (rt-PA) to reduce the incidence of tCVC malfunctions and bacteraemia [11]. At that time, rt-PA was compared with heparin locks. Later, the same authors found a significant reduction in the rate of rt-PA use for treatment of catheter malfunction using once-weekly rt-PA as a locking solution, compared with thrice-weekly heparin and citrate only [12]. Unlike the first study, there was no evidence of a significant effect on the occurrence of bacteraemia (in the risk incident and prevalent HD patients). A certain limitation of the rt-PA lock is its higher cost (a difference of CAD$962 per enrolment in the last relevant study). Based on these results we have decided to realize a prospective study (to assess the effectiveness of rt-PA once a week as a locking solution, as compared with 4% citrate only for prevention of CRBSI and catheter dysfunction) in the conditions of our local HD centre.

## Methods

The study was randomised, prospective, and double blinded. All incident patients undergoing insertion of tCVC in the Haemodialysis Centre at Charles University Teaching Hospital in Plzen were screened and included, except those with exclusion criteria: anticoagulation treatment, INR more than 1.4, platelets less than 60 × 10^9^/l, clinical symptoms of infection, known malignancy, catheter insertion in vena cava inferior region (femoral vein), bleeding complication in the four weeks before catheter insertion, major surgery in the past 48 h, major surgery planned in the next 6 months, active pericarditis and known allergy or intolerance to alteplase or trisodium citrate, and pregnancy or breastfeeding. Study participants were randomised by block method into two groups (1:1, four patients in one block) according to the solution applied as a catheter lock: 1) receiving either trisodium citrate (Citra-LockTM 4%) only or 2) rt-PA (Actilyse® 1 mg/ml, volume adjusted with saline to match the lumen) in the middle session each week with citrate used in the first and third sessions. The locks were prepared by an independent pharmacist, so both participants and medical staff were unaware of their compositions until the study ended.

To calculate the sample size, the number of patients was calculated in relation to the primary endpoint of the study, which is the incidence of CRBSI at the end of the sixth month after the insertion of the permcath. The basic hypothesis of the study is the assumption that rt-PA will lead to a reduction in the risk of CRBSI compared to sodium citrate. In the sodium citrate group, the incidence of CRBSI is assumed to be 15%. Sixty-five (power = 0.80; alpha = 0.05) patients are needed to detect a reduction in incidence of 5% or less. Due to the assumption of patient losses from follow-up, the plan is to include 80 patients. Preliminary analysis of the results with a change of protocol was not planned.

The primary outcome was the incidence of CRBSI. We modified the definition according to the Canadian definitions for catheter-related infections [13]. The definition of definite CRBSI was 1) positive blood culture from catheter and peripheral blood, and positive culture from discharge or aspirate from exit site or tunnel with identical organism, 2) positive blood culture and positive culture of catheter segment with identical organism, 3) positive blood culture and septic thrombophlebitis, or 4) positive blood culture from peripheral blood and catheter (with the identical organism) that meet the criteria for differential time to positivity (DTP) = the positivity of catheter blood culture comes at least two hours earlier. The definition of probable CRBSI was 1) two or more positive blood cultures (peripheral blood and catheter) with no evidence of source other than catheter, 2) single positive blood culture (peripheral blood or catheter) for G+ coccus with no evidence of source other than catheter, 3) strong clinical suspicion of bacterial infection with the source in the catheter (symptoms manifesting during dialysis procedure) with the necessary exclusion of other sources (urogenital and respiratory system), or 4) positive blood culture from peripheral blood and catheter (with the identical organism) that don’t meet the criteria of DTP for definite CRBSI .

The secondary outcome was the incidence of catheter malfunction/obstruction. We again modified the definition of Hemmelgarn et al. according to the K/DOQI guidelines that was defined as 1) maximal blood flow (BF) 250 ml/min or less for 30 mins or more during one dialysis procedure (max arterial and venous pressure limits of - 250 mmHg and + 250 mmHg, respectively), 2) mean BF 250 ml/min or less during two consecutive dialysis procedures (max arterial and venous pressure limits of - 250 mmHg and + 250 mmHg, respectively), 3) Reversal to catheter lines as a solution to start the dialysis with BF at least 200 ml/min (max arterial and venous pressure limits of - 250 mmHg and + 250 mmHg respectively), or 4) inability to initiate dialysis because of catheter obstruction for at least 200 ml/min, even after the reversal of catheter lines (max arterial and venous pressure limits of - 250 mmHg and + 250 mmHg respectively) [14, 15]. Treatment-related adverse effects (bleeding, hypersensitivity) were recorded. The blood flow achieved (and sustained) during the dialysis session was logged. Malfunctions were treated by application of Actilyse® 1 mg into one or both lumens according to the study protocol.

The results are presented as arithmetical means (standard errors of the mean). Statistical significance was tested with ANOVA, and post hoc analysis was performed by means of multiple linear regression. All calculations were performed using Statistica 8.0 (Stat Soft, Inc.). The study was registered at ANZCTR (ACTRN12612000152820) on the 3rd of February 2012, approved by the local Ethics Committee at Charles University Teaching Hospital in Plzen and conducted in compliance with the Declaration of Helsinki and Declaration of Istanbul. All participants were above 18 years of age and signed an informed consent form.

## Results

**Fig. 1:**
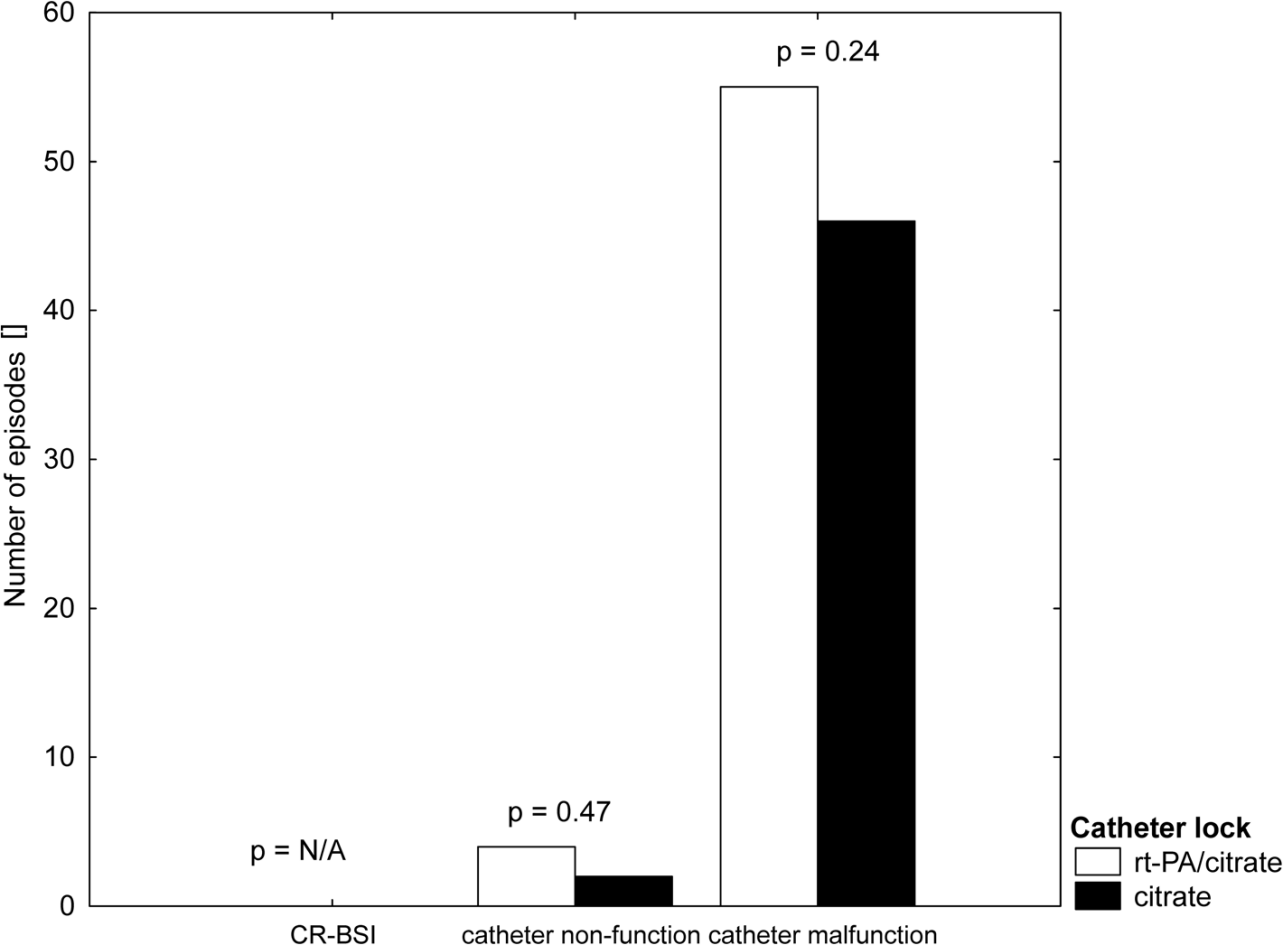
Primary (CR-BSI episodes) and secondary (catheter dysfunction) endpoints reached during the study

**Fig. 2:**
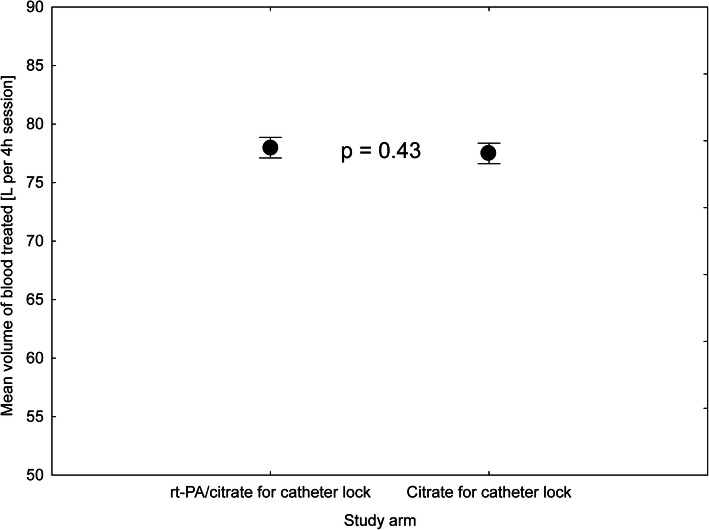
Mean volume of blood treated during HD session

**Fig. 3:**
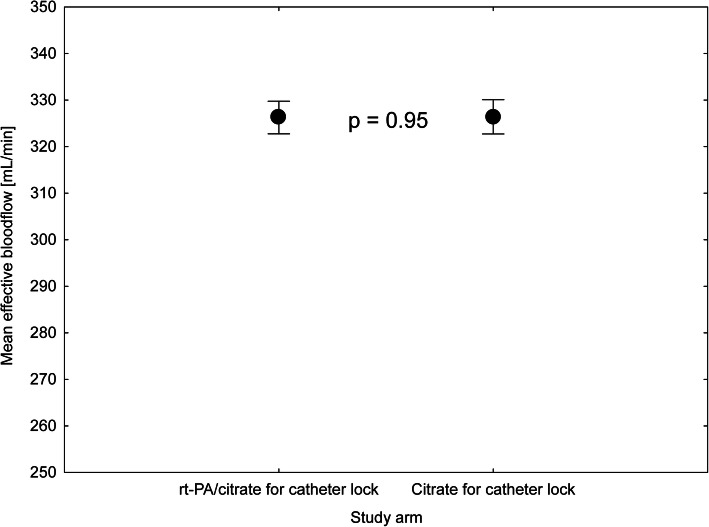
Mean effective blood flow achieved during HD session

Eighteen patients (mean age 67 ± 15.1 years, 50% males) were included and followed during 655 haemodialysis sessions between March 2012 and December 2016. The main reason for not reaching the intended numbers was an unexpected absence of CRBSI episodes and a high proportion of patients meeting the exclusion criteria. While it made analysis of the primary endpoint unfeasible (even in the extended recruitment period), evaluation of the secondary endpoint was possible. No episode of CRBSI was detected, making the incidence less than 1 in 4.3 patient years. However, it should be acknowledged that these figures may be underestimating the real occurrence due to selection bias. At the same time, six catheter non-functions (0.9% sessions) and 101 malfunctions (15.4% sessions) were recorded, and no significant difference was found comparing the group with citrate and rt-PA (Fig. 1). The incidence of both events was equal between the study groups: 4 non-functions and 55 malfunctions in the rt-PA arm and 2 non-functions and 46 malfunctions in the citrate arm (*p* = 0.47 and *p* = 0.24, respectively). Additionally, the mean blood flow achieved did not differ significantly between the groups: 326 ± 1,8 and 326 ± 1,9 ml/min (*p* = 0.95) in the rt-PA and citrate groups, respectively. Post hoc analysis identified time elapsed since previous session (β = 0.12, *p* = 0.005) and malfunction on previous session (β = 0.25, *p* < 0.001) as significant factors affecting the occurrence of malfunction (Table 1). Thus, administration of the rt-PA after the middle HD procedure did not affect the incidence of malfunction or tCVC afunction and did not affect the treated blood volume or blood flow during HD (Figs. 2 and 3).

**Table 1:** Multiple linear regression, catheter malfunction as dependent variable

Independent variables	β	Std.Err. of β	p-level
Previous malfunction [Y/N]	0,248	0,041	0,000
Interval since previous session [days]	0,122	0,044	0,005
Catheter vintage [number of sessions passed]	−0,055	0,042	0,186
rt-PA application on previous session [mg]	−0,068	0,049	0,165
Study arm [rt-PA/citrate]	0,056	0,047	0,227

Multiple linear regression: R = 0.2996 R2 = 0.0898 Adjusted R2 = 0.0814

F(5,542) = 10,690 *p* < 0.00000 Std. Error of estimate: 0.32786

## Discussion

Most valid recommendations indicate the native AVF as vascular access for chronic dialysis treatment [1–3]. However, the number of patients with tCVC is high, and depending on the source, accounts for up to 80% of patients initiating HD treatment [16–18]. The main complications associated with tCVC are infections and thrombotic occlusions [19]. The problem of patency reduces the effectiveness of dialysis, and both of these complications increase the morbidity and mortality of dialysed patients [20]. The frequency of bacteraemia is reported with rates of 2.7 per 1000 catheter days in the first month of catheter use, and 0.4 per 1000 catheter days for > 12 months [21]. The target should be a rate lower than one episode of CRBSI per 1000 catheter days [22]. In the present study, all patients were newly enrolled in the HD programme, and the bacteraemia rate of less than 0.64 per 1000 catheter days is therefore quite satisfactory. In the context of other studies, tCVCs may be considered relatively safe [11, 12]. This is in contradiction with general recommendations to avoid tCVC where possible, but as mentioned previously, for some types of people requiring dialysis, tCVC is a better alternative [23, 24]. These include, in particular, patients of advanced age with severe sclerotic lesions, often with relatively short life prospects or even considering palliative nephrology care. Under these circumstances, it is certainly not appropriate to set AVF in advance for many months, but it is better to wait for further courses. If necessary, tCVC is the best and often most lasting solution for initiating HD treatment. The results of this study, with their low incidence of CRBSI, further support this approach.

An important part of tCVC care is an aseptic approach in general and local exit site care. The aim of catheter locks is to ensure the patency of tCVC at the time of HD, and as far as possible, to reduce possible infection complications. In the context of these requirements, the composition of the locks has evolved over time. In the past, heparin was the most commonly used, but in recent years, prevalence has shifted to citrate [10]. Higher citrate concentrations (30, 46.7%) were withdrawn by FDA for possible association with serious adverse effects, and citrate locks with 4% concentration are now most commonly used [25, 26]. This solution appears to be safe, is relatively inexpensive, and has some antimicrobial activity [26, 27], which is why it is commonly recommended [28]. In the case of acute obstruction of tCVC, use of fibrinolytic agents is recommended (e.g. rt-PA [3]. Thus, the question is whether regular administration of fibrinolytic would further affect the malfunction of tCVC or the frequency of CRBSI. Because the major drawback of rt-PA is its high cost, it is usually only used once a week. When compared to heparin locks, the beneficial effects of rt-PA administration has already been shown [11]. In this study, the effect of rt-PA compared to citrate locks was verified. However, during the 655 HD procedures in 18 patients, no significant difference was found. The occurrence of malfunctions and afunctions was the same in both study groups. A linear regression found that the length of the interdialytic interval was the most important factor, specifically that the longer the interdialytic interval, the higher the risk of tCVC obstruction. At the same time, if obstruction occurs once, the risk of another obstruction is significantly higher.

Locks containing antibiotics seemed to be promising, but these are not yet generally recommended [29], mainly due to concerns about the risk of resistance. One alternative could be a lock that contains taurolidine. Taurolidine is an antimicrobial chemotherapeutic agent that acts through a chemical reaction with the microbial structure of the cell wall. It has an extremely wide microbial spectrum, including methicillin- and vancomycin-resistant bacteria, and unlike conventional antibiotics, resistance has not yet been observed [30]. However, the position of taurolidine on the field of catheter locks has to be verified by more prospective and randomised trials.

One limitation of the present study is the failure to include the required number of patients. Strict exclusion criteria eliminated most incident patients with tCVC. Another limitation was the very low occurrence of primary outcome. However, the occurrence of CRBSI was low and it can be concluded that tCVC is therefore relatively safe. It appears to be a good alternative to vascular access in patients, particularly with uncertain prognosis and exhausted vascular systems. This is in contradiction with common recommendations, but data from recent clinical practice tends to support this opinion.

## Conclusion

Regular administration of rt-PA once a week does not affect the occurrence of obstruction or malfunction of tCVC compared to citrate locks only. With the low incidence of CRBSI in the present study in general, more cannot be said about the impact on infectious complications. TCVC appears to be relatively safe and should be considered especially for the elderly population or patients with poor and uncertain prognoses, often in association with a devastated native vascular system. Until the results of larger studies with catheter locks are available, especially with taurolidine, this study suggests citrate locks are a valid recommendation.

## Funding

The study was supported by the National Sustainability Programme I [LO1503] provided by the Ministry of Education, Youth and Sports of the Czech Republic, the Charles University Research Fund [Progres Q39], and Project No. CZ.02.1.01/0.0/0.0/16_019/0000787 “Fighting Infectious Diseases “awarded by the MEYS CR, financed by EFRR.

## Data Availability

The datasets during and/or analysed during the current study are available from the corresponding author on reasonable request.

## Abbreviations

rt-PA: Recombinant tissue plasminogen activator
CR-BSI: catheter-related bloodstream infection
HD: Haemodialysis
AVF: Arteriovenous fistula
CVC: Central venous catheter
tCVC: Tunnelled central venous catheter
DTP: Differential time to positivity
BF: Blood flow
